# Analysis of direct medical expenses resulting from road traffic injuries in the City of Tabriz

**Published:** 2019-08

**Authors:** Narges Shadkam, Alireza Mahboub-Ahari, Mohammad Asghari Jafarabadi, Ali Imani

**Affiliations:** ^*a*^Department of Health Care Management, School of Management and Medical Informatics, Tabriz University of Medical Sciences, Iran.; ^*b*^Health Service Management Research Center, Department of Health Care Management, School of Management and Medical Information, Tabriz University of Medical Sciences, Tabriz, Iran.; ^*c*^Department of Statistics and Epidemiology, Faculty of Health, Tabriz University of Medical Sciences, Tabriz, Iran.; ^*d*^Health Service Management Research Center, Department of Health Care Management, School of Management and Medical Information, Tabriz University of Medical Sciences, Tabriz, Iran.

## Abstract

**Background:**

Road crashes as a major global public health problem cost 3% of most countries and 5% of low- and middle-income countries gross domestic product (GDP). The World Health Organization has predicted that without sustained action, road traffic crashes will become the seventh leading cause of death by 2030. Objectives: The aim of this study was to analyze the death rate, severity of injuries, and direct medical costs caused by road traffic injuries (RTI) in the city of Tabriz in 2014.

**Methods:**

Trauma injury admissions due to RTI in Imam Reza Hospital in Tabriz City were investigated in terms of etiology and the direct medical costs during 2014â€“2015. Data were collected using a researcher-made checklist after being confirmed by relevant experts in terms of face validity. All information on direct medical costs are extracted from several sources including hospitals, database of the Ministry of Health and Medical Education, disaster and emergency medical management center, and public and private physiotherapy clinics across the city.

**Results:**

Review of the hospital records showed that the mean age of the patients (67.9% males and 32.1% females) was 34 ± 17.3 years. In addition, 79.2% of the patients were treated on an outpatient basis, and 20.8% were treated on a hospitalization basis (hospitalization or death). The mean times of inpatient and outpatient hospitalization for injuries were 3 h and 6.7 ± 5.3 days, respectively. Total direct medical costs were 11.631 dollars, of which 8% was for hospital costs, 9.7% for pre-hospital costs, and 2.3% for physiotherapy costs. Chest and lower part injuries had highest medical costs. From etiological standpoints, the greatest reason of being injured and hospitalization is multiple injuries and bruises, and the prominent cause of death was blow to the head and neck (70%).

**Conclusions:**

The results of the present study showed that direct medical costs in Tabriz during 2014-2015 were equal to 0.1% of GDP, which is a considerable amount. High economic and social costs of road accidents and their harmful physical and psychological effects on individuals and community require the attention of professionals and experts in the transportation industry and health-care system to determine appropriate strategies for interventions in reducing accidents burden and injuries.

**Keywords:**

Road traffic injuries, Direct medical costs, Hospital, Trauma, Tabriz, Iran

